# Intestinal Epithelial-Derived TAK1 Signaling Is Essential for Cytoprotection against Chemical-Induced Colitis

**DOI:** 10.1371/journal.pone.0004561

**Published:** 2009-02-23

**Authors:** Jae-Young Kim, Rie Kajino-Sakamoto, Emily Omori, Christian Jobin, Jun Ninomiya-Tsuji

**Affiliations:** 1 Department of Environmental and Molecular Toxicology, North Carolina State University, Raleigh, North Carolina, United States of America; 2 Department of Medicine and Center for Gastrointestinal Biology and Disease, University of North Carolina, Chapel Hill, North Carolina, United States of America; New York University School of Medicine, United States of America

## Abstract

**Background:**

We have previously reported that intestinal epithelium-specific TAK1 deleted mice exhibit severe inflammation and mortality at postnatal day 1 due to TNF-induced epithelial cell death. Although deletion of TNF receptor 1 (TNFR1) can largely rescue those neonatal phenotypes, mice harboring double deletion of TNF receptor 1 (TNFR1) and intestinal epithelium-specific deletion of TAK1 (TNFR1KO/TAK1^IE^KO) still occasionally show increased inflammation. This indicates that TAK1 is important for TNF-independent regulation of intestinal integrity.

**Methodology/Principal Findings:**

In this study, we investigated the TNF-independent role of TAK1 in the intestinal epithelium. Because the inflammatory conditions were sporadically developed in the double mutant TNFR1KO/TAK1^IE^KO mice, we hypothesize that epithelial TAK1 signaling is important for preventing stress-induced barrier dysfunction. To test this hypothesis, the TNFR1KO/TAK1^IE^KO mice were subjected to acute colitis by administration of dextran sulfate sodium (DSS). We found that loss of TAK1 significantly augments DSS-induced experimental colitis. DSS induced weight loss, intestinal damages and inflammatory markers in TNFR1KO/TAK1^IE^KO mice at higher levels compared to the TNFR1KO control mice. Apoptosis was strongly induced and epithelial cell proliferation was decreased in the TAK1-deficient intestinal epithelium upon DSS exposure. These suggest that epithelial-derived TAK1 signaling is important for cytoprotection and repair against injury. Finally, we showed that TAK1 is essential for interleukin 1- and bacterial components-induced expression of cytoprotective factors such as interleukin 6 and cycloxygenase 2.

**Conclusions:**

Homeostatic cytokines and microbes-induced intestinal epithelial TAK1 signaling regulates cytoprotective factors and cell proliferation, which is pivotal for protecting the intestinal epithelium against injury.

## Introduction

The intestinal epithelium is a single cell layer that separates lamina propria immune cells from luminal components including food antigens and commensal or pathogenic bacteria [1], [2] The integrity of the intestinal epithelium is essential for the maintenance of intestinal homeostasis. The disruption of intestinal barrier results in hyper-activation of mucosal immune cells, which eventually could lead to the development of chronic inflammatory diseases such as inflammatory bowel disease (IBD) in a susceptible host. Although the etiology of IBD has not been fully elucidated, it is well known that IBD is closely associated with an abnormal response to the normal gut flora caused by the loss of intestinal barrier [3], [4]. Maintenance of this barrier depends on tight regulation of epithelial cell proliferation and apoptosis [5]. It has been demonstrated that commensal bacteria-derived signaling is important for intestinal barrier maintenance following chemical-induced injury [6]. Ablation of MyD88, a common signaling intermediate of bacteria-derived signaling pathways, causes dysregulated barrier function [6]. Commensal bacterial components can initiate MyD88 signaling in epithelial cells as well as immune cells in the intestine, both of which can modulate epithelial cell proliferation and survival. It has been well studied that homeostatic activation of immune cells is important for production of cytokines and growth factors, which critically regulate epithelial cell survival and proliferation [7]. However, because epithelial cells are constantly exposed to bacteria and several Toll-like receptors are downregulated [8], it is still unclear whether bacteria-induced signaling in epithelial cells is also important for barrier maintenance in the intestine.

Transforming growth factor-β activated kinase 1 (TAK1) is a member of the mitogen activated protein kinases kinase kinase (MAPKKK) family and plays an essential role in tumor necrosis factor (TNF), interleukin 1 (IL-1), and Toll-like receptor (TLR) signaling pathways [9], [10], [11] Furthermore, others and we have recently identified that TAK1 is a central mediator of NOD-like receptor (NLR) signaling [12], [13], [14]. In response to proinflammatory cytokines or TLR/NLR ligands, TAK1 activates both NF-κB and AP-1 pathways, which lead to activation of innate immune response. TAK1 deficiency results in embryonic lethality, therefore, conditional knockout systems have been used to reveal the in vivo roles of TAK1. It has been identified that TAK1 is required for T and B cell differentiation and their activation [10], [15], [16], [17]. Thus, TAK1 is essential for proinflammatory signaling in vivo as well as in cultured cells.

In addition to this proinflammatory function in immune cells, we have recently demonstrated that TAK1 has a completely opposite role in the skin and intestinal epithelium. TAK1 is important for preventing inflammation in the skin and intestinal epithelium [18], [19]. We reported that intestinal epithelium-specific TAK1 deleted mice (TAK1^IE^KO) spontaneously developed intestinal inflammation [18]. The TAK1-deficient intestinal epithelium underwent significant apoptosis that is induced by homeostatically expressed TNF in the intestine. The phenotype was strongly attenuated by crossing TNF receptor 1 knockout (TNFR1−/−) mice, indicating that TNF is the major cause of intestinal epithelial cell apoptosis and inflammation. However, a fraction (40–50%) of the double knockout mice (TNFR1KO/ TAK1^IE^KO) developed ileitis and colitis around the age of 14–17-days-old [18], suggesting that TAK1 is also important for preventing TNF-independent inflammation. Because TAK1 is deleted only in epithelial cells in TNFR1KO/ TAK1^IE^KO mice, this protective signal is derived from epithelial cells. As described above, TAK1 is activated by cytokines and TLR/NLR ligands. Taken together, we hypothesize that intestinal epithelial TAK1 is activated by cytokines and TLR/NLR ligands and maintains the epithelial barrier. In this study, we tested this hypothesis by analyzing TNFR1KO/TAK1^IE^KO mice. The inflammatory conditions in TNFR1KO/ TAK1^IE^KO mice at the late neonatal stage were transient and widely varied in each mouse [18]. Although some TNFR1KO/ TAK1^IE^KO mice lost weight and developed ileitis and colitis at 14–17 days as reported previously [18], more than half of TNFR1KO/ TAK1^IE^KO mice grew normally and were indistinguishable from TNFR1KO control mice after the age of 6-weeks-old. We speculate that the inflammatory conditions at 14–17 days is associated with stress caused by increased bacterial microflora in the intestine when the mice start having solid food [20]. Thus, we postulate that TAK1 signaling is important for maintenance of intestinal barrier under stress conditions. In order to study the TNF-independent role of TAK1 in the intestinal barrier, we utilized adult TNFR1KO/ TAK1^IE^KO mice and examined sensitivity to chemical-induced acute colitis. We found that TAK1 deficiency caused hypersensitivity to chemical-induced colitis involving increased apoptosis and dysregulated cell proliferation, suggesting that epithelial TAK1 signaling is cytoprotective.

## Results

### Intestinal epithelium-specific deletion of TAK1 enhances DSS-induced intestinal damage

**Figure 1:**
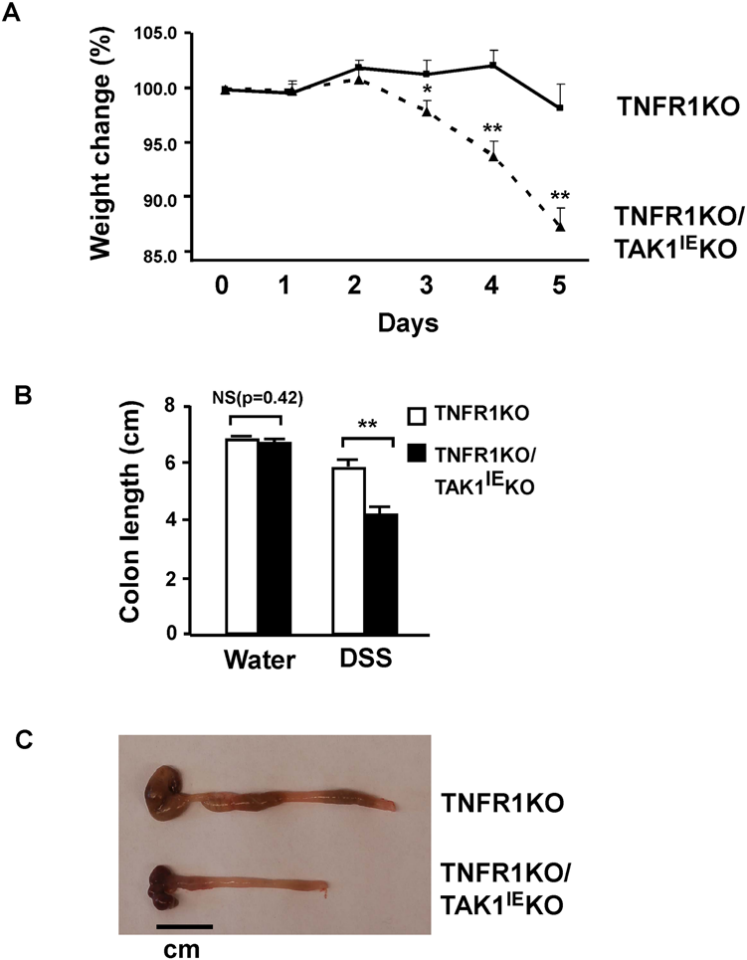
Loss of TAK1 in the intestinal epithelium results in worsened clinical signs of DSS-induced colitis. A Intestinal epithelium-specific TAK1/TNFR1 double knockout mice (TNFR1KO/TAK1^IE^KO) and control TNFR1 single knockout mice (TNFR1 KO) were fed with 2.5% DSS. Mice were weighed daily to access weight loss. Each point represents a mean value±SE. (N = 8 per group, * = p<0.05, ** = p<0.01). B Colons of TNFR1KO/TAK1^IE^KO and TNFR1 KO were removed at days 0 and 5 of 2.5% DSS administration and the colon length was measured. Each bar represents a mean value±SE (N = 6 for water control; N = 9 for DSS, ** = p<0.01, NS = not significant). C Photograph of representative colon at day 5 of DSS administration.

**Figure 2:**
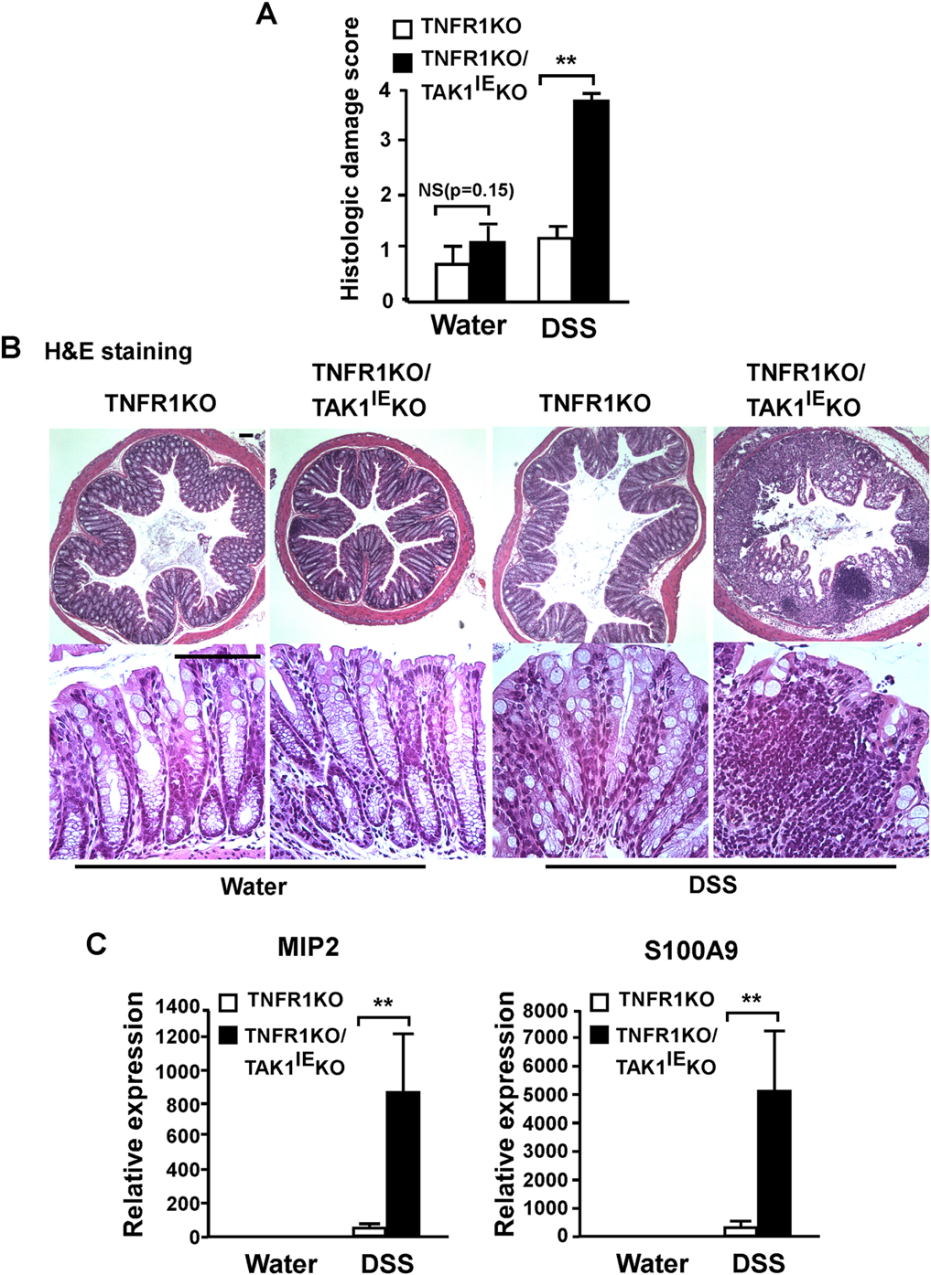
Loss of TAK1 in the intestinal epithelium leads to severe epithelial damage and upregulates inflammatory gene expression in response to DSS. A Histologic damage was scored from H&E staining sections of distal colon from TNFR1KO and TNFR1KO/TAK1^IE^KO mice treated with water or 2.5% DSS for 5 days. Each bar represents a mean value±SE (N = 6 for water control; N = 9 for DSS, ** = p<0.01, NS = not significant). B Representative H&E staining. Scale bars, 100 µm. C Total RNA was isolated from distal colon of TNFR1KO and TNFR1KO/TAK1^IE^KO mice at day 5 of DSS administration and reverse transcribed. The expression of inflammatory genes was measured using quantitative real-time PCR. Each bar represents a mean value±SE (N = 5 per group, ** = p<0.01).

In order to investigate the hypothesis that epithelial-derived TAK1 signaling is important for maintenance of intestinal barrier under stress conditions, we exposed adult TNFR1KO/ TAK1^IE^KO mice to dextran sulfate sodium (DSS), a chemical disrupting the epithelium. TNFR1KO/TAK1^IE^KO and littermate control TNFR1KO mice were fed with 2.5% of DSS and sacrificed at Day 5. The severity of DSS-induced colitis was monitored by daily loss of body weight and observation of clinical signs of acute inflammation such as rectal bleeding. DSS treatment caused little weight loss or no apparent injury phenotype in the control TNFR1KO mice. In contrast, the double mutant TNFR1KO/TAK1^IE^KO mice showed significant weight loss and rectal bleeding starting from Day 4 (Fig. 1A and data not shown). DSS-induced colon shortening, a marker for intestinal damage, was also observed in TNFR1KO/TAK1^IE^KO mice (Fig. 1B and C). To further evaluate the role of TAK1 in DSS-induced intestinal injury and inflammation, histological and biochemical analyses were performed. Without DSS treatment, the colon from TNFR1KO/TAK1^IE^KO showed only slight signs of inflammatory conditions and was almost indistinguishable from TNFR1KO control mice (Fig. 2A and B). After 5 days of DSS treatment, TAK1-deficient colon underwent complete loss of crypt architecture and severe ulceration, whereas control mice showed only mild damages characterized by epithelial hyperplasia and immune cell infiltration (Fig. 2A and B). The structural damages in TAK1-deficient colon might be associated with severe inflammation. To verify this possibility, the expression levels of inflammatory genes were measured. After 5 days of DSS treatment, the distal colons were isolated and the mRNA levels of chemokine MIP2 (IL-8 in human) and chemotactic factor S100A9 were determined by the real-time PCR. Both MIP2 and S100A9 expression was greatly upregulated in TAK1-deficient colon, presumably due to an increased activity of infiltrated immune cell (Fig. 2C). These data indicate that TAK1 signaling in the intestinal epithelium protects against DSS-induced intestinal injury and acute colitis.

### TAK1 is required for intestinal epithelial cell survival following DSS exposure

**Figure 3:**
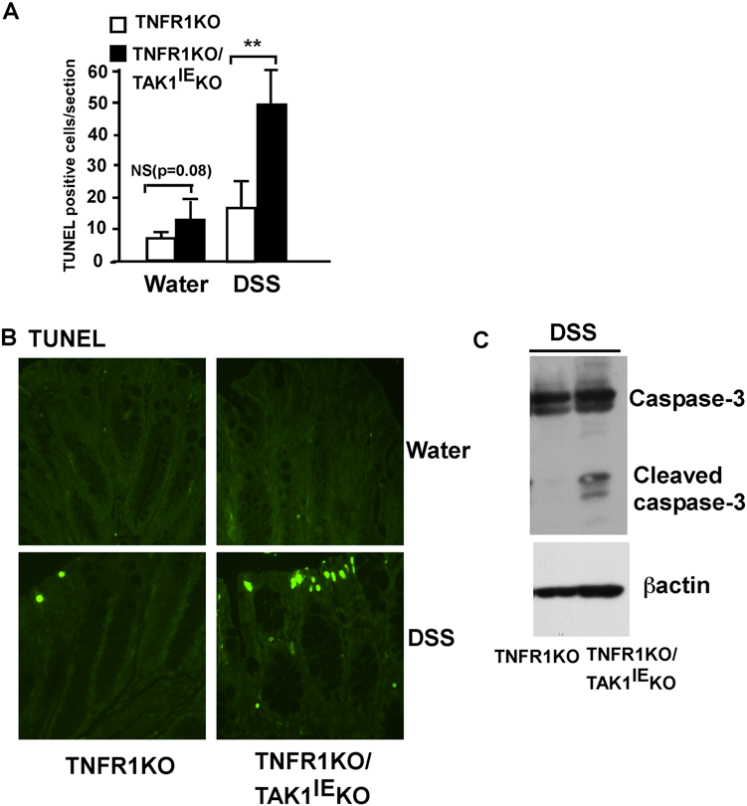
TAK1 protects the intestinal epithelium from apoptosis in response to DSS-induced damage. A Distal colon was isolated from TNFR1KO and TNFR1KO/TAK1^IE^KO mice treated with water or 2.5% DSS for 3 days. To detect apoptotic cells, TUNEL staining was performed and TUNEL positive cells were counted. Each bar represents a mean value±SE (N = 4 for water control; N = 5 for DSS, ** = p<0.01, NS = not significant). B Representative TUNEL staining. C Distal colon was isolated from TNFR1KO and TNFR1KO/TAK1^IE^KO mice treated with 2.5% DSS for 3 days. Cell extract was isolated and subjected to immunoblotting using caspase-3 antibody. β-actin was used for loading control.

In order to protect the intestinal epithelium from DSS-induced injury, two biological processes are essential; one is suppression of apoptosis; and another is cell proliferation to repair lesions. We first examined apoptosis in TNFR1KO/TAK1^IE^KO and control TNFR1KO mice. In the DSS colitis model, these biological events precede clinical signs such as weight loss or rectal bleeding. Therefore, we examined apoptosis in colonic epithelial cells in mice exposed to DSS for 3 days, a time point preceding clinical signs of colitis (Day 4) in the double mutant mice (Fig. 1A). TUNEL staining was performed to assess apoptosis, and the results revealed that DSS exposure induced more pronounced apoptosis in the TAK1-deficient intestinal epithelium compared to the control mice (Fig. 3A and B). In order to confirm this data, the distal colon was isolated from DSS treated mice and the proteins lysates were analyzed for caspase-3 processing using Western blot. Consistent with the TUNEL result, the amount of cleaved caspase-3 was greatly increased by the administration of DSS in the TAK1-deficient colon, but not in control colon (Fig. 3C). These data indicate that TAK1 is required for suppression of apoptosis against DSS-induced intestinal damage.

### Loss of TAK1 dysregulates proliferation of the intestinal epithelium

**Figure 4:**
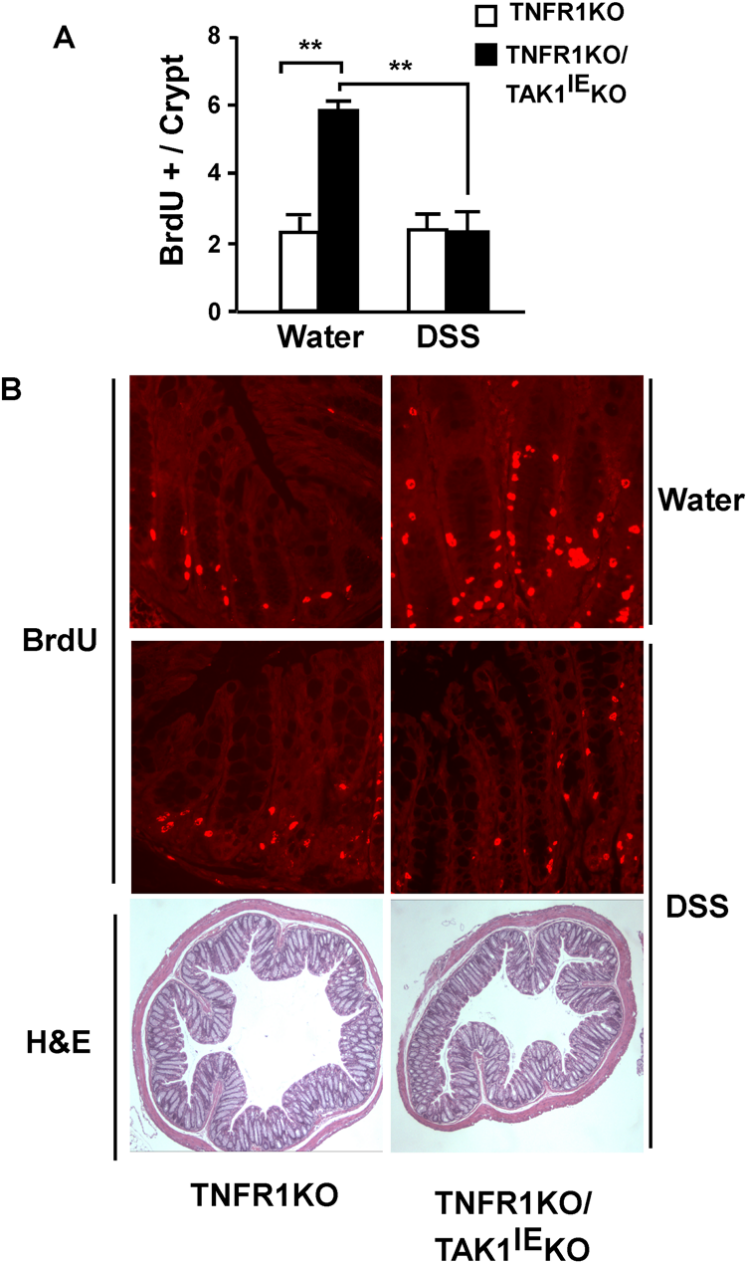
Loss of TAK1 disrupts homeostatic proliferation of the intestinal epithelium. A Distal colon was isolated from TNFR1KO and TNFR1KO/TAK1^IE^KO mice treated with water or 2.5% DSS for 3 days. Mice were injected with 0.1 mg/g of 5-bromo-2′-deoxyuridine (BrdU) 2 h prior to sacrifice. Immunostaining for BrdU was performed and BrdU-positive cells were counted. Each bar represents a mean value±SE (N = 5 per group, ** = p<0.01). B Representative BrdU staining and H&E staining at 3 days DSS treatment. Original magnifications; ×200 (BrdU staining) and ×40 (H&E staining).

We next examined epithelial cell proliferation. Intestinal homeostasis is maintained by proliferation and differentiation of the intestinal epithelial cells along the crypt axis. It has been reported that commensal bacteria-mediated TLR signaling is important for homeostatic level of proliferation of epithelial cells. Depletion of commensal bacteria or loss of TLR signaling abnormally upregulates the steady-state level of epithelium proliferation and lead to increased sensitivity to chemical and radiation-induced intestinal injury [6]. TAK1 is a key mediator of bacteria-induced signaling pathways, TLR/NLR signalings. Thus, we hypothesize that TAK1-deficient intestinal epithelial cells do not respond to commensal bacteria, which may dysregulate cell proliferation along the crypt axis. This may be associated with increased sensitivity to DSS-induced injury. To address this possibility, we examined the base line proliferating status in the intestinal epithelium of control TNFR1KO and TNFR1KO/TAK1^IE^KO mice. Proliferating cells were labeled by 5-bromo-2′-deoxyuridine (BrdU) that was injected 2 h prior to harvesting distal colon. Immunohistochemical analysis was performed to detect BrdU labeled cells. Interestingly, we found significantly increased proliferating cells in TAK1 deficient intestinal epithelium (Fig. 4A and B). Of note, whereas proliferating cells were restricted in the bottom regions of the crypts (stem cell area) in control TNFR1KO mice, a large fraction of proliferating cells was found in the middle or upper region of the crypts in the TNFR1KO/TAK1^IE^KO mice. It is known that DSS-induced injury induce compensatory cell proliferation responses in mice [6]. Mice were fed with 2.5% DSS for 3 days and proliferating cells were examined by BrdU. Cell proliferation was not altered by DSS treatment in control TNFR1KO mice (Fig. 4A and B), which is consistent with the results showing no severe injury in the control mice under 2.5% DSS treatment (Fig. 1). In TNFR1KO/TAK1^IE^KO mice, the length and structure of crypts were still relatively intact at 3 days with 2.5% DSS treatment (Fig. 4B, H&E staining). However, we found a dramatic reduction of cell proliferation in TAK1-deficient intestinal epithelium after DSS exposure (Fig. 4A and B, BrdU staining). This indicates that reparative cell proliferation is impaired in TAK1-deficient epithelial cells. Taken together, intestinal epithelium-specific TAK1 deficiency results in the disruption of homeostatic epithelial cell proliferation and impairs compensatory epithelial cell proliferation after DSS-induced injury, which might be responsible for greater sensitivity to the injury-mediated inflammation.

### TAK1 is required for proinflammatory cytokine- and bacterial components-induced cytoprotective gene expression

**Figure 5:**
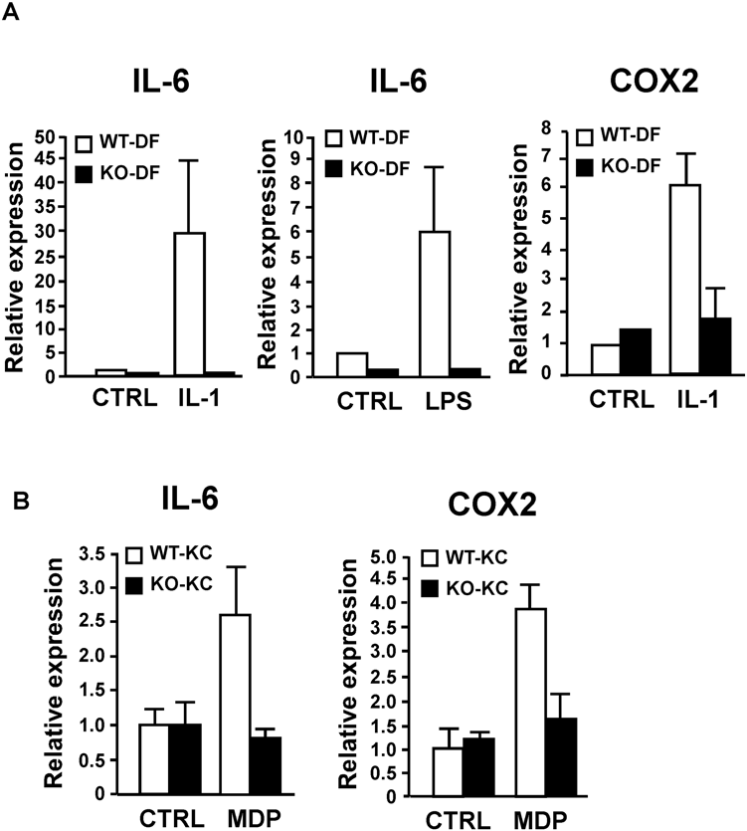
TAK1 is required for the expression of protective factors in response to proinflammatory cytokine or bacterial components. A TAK1 WT and KO dermis fibroblasts (DF) were stimulated with 5 ng/ml of IL-1β or 2 µg/ml of LPS for 6 h. The expression of IL-6 and COX2 was examined by quantitative real-time PCR. mRNA levels were normalized with the levels of GAPDH. Relative mRNA levels were calculated using those of untreated TAK1 WT dermis fibroblasts. Data are mean±S.E. of three independent samples and representative of three independent experiments with similar results. B TAK1 WT and KO keratinocytes (KC) were stimulated with 10 µg/ml of MDP for 6 h. The expression of IL-6 and COX2 was examined as described above.

We next investigated the mechanism by which epithelial TAK1 signaling protects against DSS-induced injury. We examined activation of TAK1 pathway, cell proliferation and cell survival in cultured cells. Because primary culture of intestinal epithelial cells is technically difficult due to detachment-induced apoptosis (anoikis) during the isolation procedure [21], we utilized intestinal epithelial cell lines, keratinocytes and dermis fibroblasts as model systems. We first examined whether DSS affects TAK1 signaling including activation of NF-κB and MAPK pathways. We found that DSS barely activated NF-κB or MAPKs in the intestinal epithelial cell lines including HT-29 and Caco2 as well as keratinocytes (data not shown). We next examined whether TAK1 deletion alters the cell proliferation and survival using wild-type and TAK1-deficient cells. We found that TAK1-deficient keratinocytes and fibroblasts did not show any impaired cell proliferation or increased cell death upon DSS treatment (data not shown). These indicate that DSS does not directly activate TAK1 pathway, and that TAK1 signaling does not cell-autonomously regulate cell death or proliferation in response to DSS exposure. Based on these findings, we postulate that epithelial TAK1 is activated not by DSS but by cytokines and TLR/NLR ligands that are upregulated by DSS-induced injury, and that TAK1 signaling may induce production of cytoprotective factors and facilitate epithelial cell survival and proliferation. To address this hypothesis, we tested whether TAK1 is required for expression of cytoprotective factors, interleukin 6 (IL-6) and cycloxygenase 2 (COX2), following exposure of activators of TAK1 signaling, IL-1, LPS and the NOD2 ligand, muramyl dipeptide (MDP). We found that TAK1 is required for IL-1-induced IL-6 and COX2 expression in fibroblasts (Fig. 5A). LPS also induced IL-6 expression in a TAK1 dependent manner in fibroblasts (Fig. 5A). In wild type keratinocytes, both IL-1 and LPS failed to induce IL-6 and COX2 expression (data not shown), because keratinocytes do not strongly respond to LPS and IL-1 stimulation. However, MDP strongly upregulated the levels of IL-6 and COX2 in wild type keratinocytes, and a process completely abolished by the loss of TAK1 (Fig. 5B). These data suggest that epithelial TAK1 signaling is essential for production of cytoprotective factors.

## Discussion

In this study, we demonstrated that intestinal epithelium-derived TAK1 signaling plays a pivotal role in preventing injury-induced intestinal inflammation. In the absence of TAK1-derived signaling, the intestinal epithelium is exquisitely sensitive to DSS-induced injury, a process involving increased epithelial cell apoptosis and reduced regenerative proliferative responses. This pathologic response further induces damage-associated inflammation. In addition, ablation of TAK1 leads to an increased expansion of the epithelial cell proliferative zone under steady-state conditions, and causes impaired reparative proliferation after injury. Finally, we showed that loss of TAK1 abolishes production of cytoprotective factors in response to proinflammatory cytokines and bacterial components. Taken together, we propose that TAK1 is essential for preventing injury-associated intestinal inflammation by the following two mechanisms. One is that TAK1 is responsible for maintenance of homeostatic proliferation of the intestinal epithelium, and that dysregulated cell proliferation causes hypersensitivity to cytotoxic insults. Intestinal epithelial cells are originated from stem cells located in the bottom of crypts [2]. The intestinal stem cell-derived epithelial cells are proliferating until terminally differentiated. The population of these proliferating cells is tightly regulated. We found that the steady-state level of proliferation is abnormally upregulated in the TAK1-deficient intestinal epithelium. Although increased proliferating cells may be beneficial for tissue repair, proliferating cells are usually hypersensitive to cytotoxic insults. It is well exemplified in the case of cancer therapy. The gastrointestinal tract, oral mucosa and hematopoietic cells, which are all highly proliferative, are the most sensitive to chemotherapy. Another mechanism is that epithelial TAK1 signaling induces cytoprotective factors, and that ablation of this pathway causes increased apoptosis and degeneration of the intestinal barrier. We found that TAK1 is essential for IL-1, LPS and MDP-induced expression of IL-6 and COX2, both of which are known to play an important role in protecting the intestinal epithelium against DSS-induced injury [22], [23], [24], [25]. Commensal bacteria and homeostatic cytokines activate TAK1 in the intestinal epithelium, and the activated TAK1 induces cytoprotective factors including IL-6 and COX2. We propose that this pathway is essential for the maintenance of intestinal barrier integrity.

TAK1 can activate two major transcription factors, NF-κB and AP-1. NF-κB is a key transcription factor implicated in the regulation of both IL-6 and COX2 gene expression [26]. Pharmacological blockage of NF-κB inhibits production of COX2 in intestinal epithelial cells and enhances DSS-induced colitis [23]. Therefore, TAK1-NF-κB signaling pathway in intestinal epithelial cells may be the major pathway for maintenance of the intestinal barrier. However, intestinal epithelial-specific NEMO (IKKγ) deletion mice, which is deficient in NF-κB activation, develop TNF-dependent but not TNF-independent intestinal inflammation [27]. Therefore, whereas TAK1-NF-κB pathway participates in prevention of TNF-induced cell death and inflammation, TAK1-dependent but NF-κB-independent pathways are also important for the intestinal barrier function.

How does TAK1 deletion cause increased basal cell proliferation in the intestinal epithelium? It has been established that proliferation of the intestinal epithelium depends on the concerted action of several factors including Wnt (positive regulator of cell cycle) and TGF-β signaling (negative regulator of cell cycle) [2], [28], [29], [30]. TAK1 is involved in both Wnt (negatively) and TGF-β (positively) signaling pathways in cultured cells [31], [32], [33], [34]. Therefore, TAK1 deletion may affect epithelial cell proliferation through dysregulating Wnt or TGF-β signaling. However, we note that intestinal epithelial-specific deletion of TAK1 does not alter epithelial cell proliferation and differentiation at least during embryogenesis [18]. Therefore, TAK1 is not a major signaling intermediate of Wnt and TGF-β signaling in the intestinal epithelium. Besides Wnt and TGF-β signalings, intestinal epithelial proliferation is regulated by innate immune signaling [6]. MyD88-deficient mice show increased proliferation in the intestinal epithelium [6], which is similar to our TNFR1KO/TAK1^IE^KO mice. It has been reported that NF-κB p50-deficient intestinal epithelium also shows extensive proliferative zones [35], and that NF-κB signaling has been reported to inhibit cell proliferation in epithelial tissues [36]. TAK1 is a signaling intermediate of MyD88-NF-κB pathway [37]. Therefore, MyD88-TAK1-NF-κB pathway may function to limit the proliferative zones in the intestinal epithelium by preventing epithelial cell proliferation.

TLR- or MyD88-deficient mice and commensal bacteria-depleted mice showed very similar phenotypes observed in our TNFR1KO/TAK1^IE^KO mice that are characterized by increased susceptibility to DSS-induced injury, dysregulated steady-state levels of cell proliferation and impaired production of cytoprotective factors [6]. Rakoff-Nahoum et al. proposed that commensal bacteria-induced signaling is important for maintenance of the intestinal barrier. Failure to initiate activation of these signaling pathways increases susceptibility of the intestinal epithelium to chemical or radiation-induced injury. However, because these studies have used germline knockout of TLR4, TLR2 and MyD88, it is not clear whether epithelial cell- or immune cell-derived signaling is responsible for maintaining barrier integrity. Our results demonstrated that epithelial-derived TAK signaling is important for the maintenance of intestinal homeostasis. It should also be of interest to determine the contributions of immune cell-derived TAK1 signaling to the barrier maintenance.

Loss of intestinal epithelial barrier is associated with chronic inflammatory diseases such as inflammatory bowel diseases (IBD). Mutations in NOD2 gene are highly associated with IBD susceptibility [3]. Our results indicate that TAK1 signaling is essential for epithelial barrier maintenance. TAK1 is a key mediator of NOD2 signaling [14]. Taken together, loss of NOD2-TAK1 signaling in the intestinal epithelium may be one of the causes to increase susceptibility of IBD. However, further investigation will be needed to clarify the relationship between TAK1 and the pathogenesis of IBD.

## Materials and Methods

### Mice and Induction of DSS Induced-Colitis

TNFR1KO/TAK1^IE^KO mice and control *TAK1^FL/FL^ TNFR1* ^−/−^ were generated as described in our previous study [18]. All mice were on a C57BL/6 background and genotypes were determined by PCR. For inducing acute colitis, 6 to 8 weeks-old TNFR1KO/TAK1^IE^KO mice and littermate control TNFR1KO (*TAK1^FL/FL^ TNFR1* ^−/−^) were fed with 2.5% of DSS. Mice were weighed daily and sacrificed at time points indicated. Mice were bred and maintained under specific pathogen-free conditions. All animal experiments were done with the approval of the North Carolina State University Institutional Animal Care and Use Committee.

### Cells

Dermis fibroblasts were isolated from TAK1*1^FL/FL^* mice. The cells were spontaneously immortalized and infected with pMX-puro-CRE retroviral vector to delete *tak1* gene. Uninfected cells were removed by puromycin selection and the deletion of TAK1 was confirmed by immunoblotting. The cells were maintained in DMEM containing 10% bovine growth serum (HyClone) and 1% penicillin/streptomycin. TAK1 WT and KO keratinocytes were isolated from epidermis-specific TAK1 deletion mice and maintained in keratinocyte medium as described in our previous publication [19]

### Histology and Immunohistochemical analysis

After 5 days of DSS treatment, distal colons were isolated and fixed in 10% formalin. Paraffin-embedded sections were stained with H&E for histological analysis. Sections were scored in a blinded fashion on a scale from 0 to 4, based on the degree of lamina propria mononuclear cell infiltration, crypt hyperplasia, goblet cell depletion, and architectural distortion, as previously described [38]. Apoptotic cells were detected after 3 days of DSS treatment using the DeadEnd colorimetric TUNEL system (Promega) according to the manufacturer's instructions. To detect proliferating cells, mice were injected with 0.1 mg/g of BrdU 2 h prior to sacrifice. Immunohistochemical analysis was performed using anti-BrdU antibody (Becton Dickinson).

### Real-time PCR analysis

Total RNA was prepared from distal colons or cells using RNeasy minikit (Qiagen). To obtain cDNA, 200 ng of each RNA samples were reverse-transcribed using TaqMan reverse transcription reagents (Applied Biosystems). Real-time PCR analysis was performed using the ABI PRISM 7300 sequence detection system (Applied Biosystems). All samples were normalized by the amount of glyceraldehyde-3-phosphate dehydrogenase (GAPDH) expression.

### Immunoblotting

After DSS exposure, distal colons were harvested and homogenized in an extraction buffer (20 mM HEPES (pH 7.4), 150 mM NaCl, 12.5 mM β-glycerophosphate, 1.5 mM MgCl_2_, 2 mM EGTA, 10 mM NaF, 2 mM DTT, 1 mM Na_3_VO_4_, 1 mM PMSF, 100 U/ml aprotinin, 0.5% Triton X-100). Proteins from cell lysates were electrophoresed by SDS-PAGE and transferred to Hybond-P (GE Healthcare). The membranes were immunoblotted with a polyclonal antibody against caspase-3 (Cell signaling) and a mouse antibody against β-actin (Sigma-Aldrich). Bound antibodies were visualized with HRP-conjugated antibodies against rabbit or mouse IgG using the ECL Western blotting system (GE Healthcare).

### Statistical analysis

Statistical comparisons were made using paired or independent two-tailed Student's t tests assuming equal variance and two-tailed Welch's t tests assuming unequal variance.
